# Supporting Employment After Cancer: A Mixed-Methods Evaluation of a Vocational Integration Programme for Childhood, Adolescent, and Young Adult Cancer Survivors

**DOI:** 10.3390/curroncol32100564

**Published:** 2025-10-08

**Authors:** Margherita Dionisi-Vici, Anna Schneider-Kamp, Ilenia Giacoppo, Alessandro Godono, Eleonora Biasin, Antonella Varetto, Emanuela Arvat, Francesco Felicetti, Giulia Zucchetti, Franca Fagioli

**Affiliations:** 1 Clinical Psychology Unit, Azienda Ospedaliero Universitaria Città della Salute e della Scienza Hospital, 10126 Turin, Italy; margheritadionisivici@gmail.com (M.D.-V.); avaretto@cittadellasalute.to.it (A.V.); 2 U.G.I. Association, Unione Genitori Italiani Contro Il Tumore dei Bambini, Corso Dante 101, 10126 Turin, Italy; 3 Department of Business and Management, University of Southern Denmark, 5230 Odense, Denmark; anna@sam.sdu.dk; 4 Pediatric Oncology Division, Regina Margherita Children’s Hospital, Azienda Ospedaliero Universitaria Città della Salute e della Scienza Hospital, 10126 Turin, Italy; ilenia.giacoppo@gmail.com (I.G.); eleonora.biasin@unito.it (E.B.); franca.fagioli@unito.it (F.F.); 5 Department of Public Health and Pediatric Sciences, University of Turin, 10126 Turin, Italy; alessandro.godono@unito.it; 6 Oncological Endocrinology, Azienda Ospedaliero Universitaria Città della Salute e della Scienza Hospital, 10126 Turin, Italy; emanuela.arvat@unito.it (E.A.); ffelicetti@cittadellasalute.to.it (F.F.); 7 Department of Medical Sciences, University of Turin, 10126 Turin, Italy

**Keywords:** CAYAC survivors, vocational integration programme, intervention, late effects, health related quality of life

## Abstract

**Simple Summary:**

Childhood, adolescent, and young adult cancer (CAYAC) survivors often face difficulties entering the workforce, and this transition is frequently complicated by the long-term effects of illness and treatment. Although guidelines emphasise the importance of vocational support, few structured interventions have been developed and systematically evaluated for this population. This study explored a vocational integration programme specifically designed for CAYAC survivors in a tertiary cancer centre in Italy. The programme combined individualised career guidance, soft skills training, and a paid internship, delivered by a multidisciplinary team. A mixed-method approach was used to examine both feasibility and impact, providing a comprehensive understanding of how such interventions may respond to the complex needs of young cancer survivors. The study highlights the significance of tailored, socially supportive vocational programmes and offers insights that may inform future research, clinical practice, and policy development in the field of survivorship care.

**Abstract:**

Childhood, adolescent, and young adult cancer (CAYAC) survivors often face challenges entering the workforce due to long-term physical, cognitive, and psychological late effects, defined as chronic health conditions resulting from cancer and its treatments. This study evaluated a vocational integration programme that addresses these barriers and promotes psychosocial well-being. The multidisciplinary intervention combined career guidance, soft-skills training, and a paid internship. Using a mixed-method design with questionnaires and semi-structured interviews, we assessed feasibility, satisfaction, and psychosocial outcomes. Thirteen participants (mean-age-at-diagnosis: 12.9 years, SD 5.2; mean-age-at-interview: 27.2 years, SD 5.3) reported over 40 late effects, mostly of moderate severity. Health-Related Quality of Life (HRQoL), measured by the SF-12, showed a Physical Component Score mean of 45.2 (SD 9.1) and a Mental Component Score mean of 43.5 (SD 11.2), indicating greater psychological impact. The programme received high satisfaction ratings (mean 8.3/10) and was described as motivating and valuable, enhancing self-confidence and career prospects. Social support emerged as a key facilitator, while participants noted the need for flexibility and individualised pacing. Despite a limited sample size and potential recruitment bias, this study provides preliminary insights into the feasibility and perceived value of tailored vocational programmes, emphasising the importance of adaptable, socially supportive interventions for CAYAC survivors.

## 1. Introduction

Recent improvements in therapeutic strategies for the treatment of childhood, adolescent, and young adult cancers (CAYAC) have significantly enhanced survival outcomes, with current 5-year survival rates reaching over 80% [1,2]. As a result, the distinctive population of CAYAC survivors continues to grow steadily, presenting a unique set of medical, psychological, and psychosocial needs [1,2,3,4,5,6].

Depending on the type of treatment received, CAYAC survivors are at increased risk of developing late effects (LE), a wide range of chronic or delayed health conditions that appear months or years after the completion of cancer therapies, requiring long-term monitoring through specialised and multidisciplinary follow-up care. Timely prevention and monitoring facilitate early detection and limit adverse outcomes [2,7,8]. These sequelae may include physical and neurocognitive impairments, mental health difficulties, psychosocial challenges, and an elevated risk of relapse or second primary tumours [9,10,11,12,13].

One critical concern during the transition of CAYAC survivors to adulthood is the impact of their medical history on social and occupational circumstance [14]. Educational achievements and employment outcomes are key indicators of social functioning and personal independence during adulthood [15]. However, compared with the general population, CAYAC survivors are at higher risk of unemployment, underemployment, health-related job absenteeism, and difficulties entering or maintaining full-time work, contributing to prolonged dependence and reduced financial autonomy [6,16,17,18,19,20,21].

Central nervous system (CNS) tumours, secondary disease or relapse, neurocognitive and sensory impairments, and treatment exposure such as cranial irradiation, neurosurgery, amputations, or specific chemotherapies (e.g., alkylating agents, vincristine) have been recognised as risk factors for poor occupational outcomes. Mental health vulnerability and socio-demographic factors, such as female gender and low educational attainment, could further increase this vulnerability [6,15,18,21,22,23,24].

Based on these findings, recent evidence-based guidelines [5,11,21,24,25,26] have emphasised the need to incorporate occupational monitoring and support within multidisciplinary follow-up care for CAYAC survivors. Specifically, vocational planning and employment surveillance should be initiated already in late adolescence, particularly during the transition from school to the workforce. A multidisciplinary team approach should be implemented, enabling the referral of survivors to specialised professionals when specific challenges are identified in the area of employment. Such professionals may include vocational counsellors, psychologists experienced with the CAYAC survivor’s population, or social workers. This approach allows for timely assessment and the implementation of tailored services to improve occupational outcomes and overall quality of life for CAYAC survivors.

Despite existing recommendations, practical interventions remain underreported in the literature [21,27]. Available studies are heterogeneous in aims and methodologies [28]. Some have focused on psychosocial support to improve well-being and coping skills [29,30,31], while others specifically address return-to-work (RTW) strategies for adult cancer survivors [32], typically involving workplace modifications. Only one intervention adopted an integrated care model, and very few targeted physical workload or work ability. Zegers et al. [33] highlighted the greater effectiveness of multidisciplinary, personalised support tailored to an individual’s state of motivation. However, these approaches are designed for adults returning to employment and may not apply directly to CAYAC survivors, who often face the challenge of entering the workforce for the first time. In Canada, a promising example of individualised support for childhood cancer survivors is the School and Work Transitions Program (SWTP), which used Goal Attainment Scaling to set and monitor personalised educational and vocational goals, demonstrating the value of tailored interventions in promoting first-time workforce integration [34].

Adapting such approaches may be particularly valuable for CAYAC survivors, supporting their first steps into employment through individualised, structured interventions aimed at autonomy and long-term integration, especially for those with significant functional or psychological limitations due to their prior illness [15,25].

The primary aim of this study is to describe the experience of implementing such recommendations through a multidisciplinary work placement intervention, designed for CAYACs who have survived paediatric cancer and based in Turin, Italy.

Using a mixed-methods follow-up study combining a validated questionnaire with qualitative semi-structured interviews, the study explores participants’ perceptions of the intervention’s effectiveness and impact, with particular regard to perceived barriers and facilitators to employment integration and long-term occupational outcomes.

## 2. Materials and Methods

### 2.1. The Intervention

In Turin (Italy), since 1980 [35], the Italian association Parents’ Union Against Childhood Cancer (Unione Genitori Italiani contro il Tumore dei Bambini, UGI) has collaborated with the Paediatric Hemato-Oncology Department of the Regina Margherita Children’s Hospital, and later also with the Transition Unit for Paediatric Cancer Survivors, promoting initiatives aimed at supporting the well-being of paediatric oncology patients both during and after their illness.

Since 2021, among other initiatives, the UGI association has implemented targeted actions to support the employment of young adults treated for childhood cancer. These projects are carried out in collaboration with Patchanka [36], a social cooperative and employment agency (accredited by the Piedmont Region), which offers support to unemployed individuals—particularly those facing disadvantage—through career guidance and vocational redevelopment services.

This partnership has enabled the development of specialised pathways for beneficiaries who have been left with temporary or permanent disabilities following cancer treatment, applying specific ‘Place and Train’ methodologies in which Patchanka staff are trained.

Each year, approximately 4–5 CAYAC survivors join the project. They are engaged in a personalised pathway lasting around 12 months and comprising several phases: orientation, a paid internship of approximately six months in a company, and subsequent support either for direct employment or further job searches.

The team is composed of a psychologist specialised in CAYAC survivorship, a UGI representative, and Patchanka job placement specialists. When necessary, consultation with an occupational physician may also be requested for assessments or certifications.

**Figure 1:**
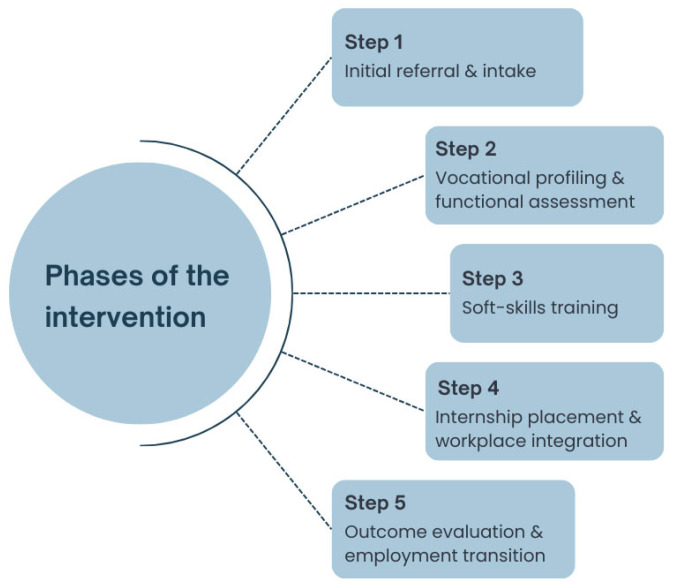
The five phases of the vocational intervention for CAYAC survivors: enrolment, assessment, orientation, training, and internship.

Participants are identified through various channels: personal requests, follow-up medical visits, rehabilitation sessions, or via association volunteers. No strict exclusion criteria are applied—any unemployed adult CAYAC survivor experiencing employment-related difficulties connected to previous cancer is eligible for enrolment on the project and, subsequently, the follow-up study. The intervention consists of five phases, which are illustrated in Figure 1.

1. Initial referral and Intake: The beneficiary is connected with the multidisciplinary team and referred to the designated Patchanka project manager.
2. Vocational profiling and functional assessment: An initial assessment phase is conducted, involving individualised vocational guidance, curriculum vitae development, functional and disability evaluations, and mapping of skills, interests, and employment-related barriers.
3. Soft-skills training: A subset of beneficiaries engages in group sessions through theatrical techniques via the JobAct^®^ Method, aimed at enhancing soft skills such as communication, emotional regulation, and workplace self-efficacy [37].
4. Internship Placement and Workplace Integration: Patchanka employment specialists identify host companies compatible with the physical, cognitive, and professional profiles of participants. They provide structured support throughout workplace integration, including onboarding facilitation and interpersonal mediation. The internship is remunerated and fully funded by UGI. Participants proceed autonomously while retaining access to the project manager for case-specific support.
5. Outcome Evaluation and Employment Transition: Upon completion of the internship, outcome evaluations are performed. These may lead to an extension of the placement, direct employment, or the initiation of new job-matching procedures.

### 2.2. The Mixed-Methods Follow-Up Study

The sampling frame for the follow-up study are CAYAC survivors in Turin that participated in a project/intervention aimed at workplace integration and/or increasing employability. Given this inherently limited sampling frame and the expected diversity and complexity of the experiences and perceptions of the participants, we opted for a non-sequential predominantly qualitative [38] mixed-methods design combining a validated questionnaire with qualitative semi-structured interviews.

Participants were recruited through telephone calls by either the project psychologist or members of the Patchanka team. After explaining the objectives of the study and ensuring participants’ understanding, written informed consent was obtained prior to participation. The interviews were conducted via video calls between May and July 2025.

The purpose of the questionnaire is to assess the self-reported health status of the participants (Health related quality of life, HRQoL). To this end, we employ the validated and well-established SF-12 (12-Item Short Form Health Survey) questionnaire [39], which provides two composite indices: the Physical Component Summary (PCS) and the Mental Component Summary (MCS). Both scores are standardised, with values > 50 indicating better-than-average physical or mental health, and scores < 50 suggesting poorer health status.

For the qualitative semi-structured interviews, we followed state-of-the-art best practices [40] and developed a thematic interview guide covering the themes of interview and participant information, orientation phase participation, internship experience, integration and professional support, accessibility and physical suitability, current employment situation, and personal impact and social support.

The participants were sampled purposely for maximum variation from the participants of several initiatives aiming at integrating childhood cancer survivors into workplace settings and increasing their downstream employability. Within the confines of the limited sampling frame, we strove for representing a variety of diagnoses and age groups (both age-at-diagnosis and age-at-interview). We iteratively and continuously analysed the interviews, measuring the degree of data saturation based on the extent to which the most recent interview supported rather than extended existing categorisations.

All interviews were described verbatim in Italian and subsequently translated equivalently and adequately to English for analysis. We employed reflexive thematic analysis [41] relying on an inductive approach, starting with open codes, collecting these into categories, and connecting and condensing these categories into themes. Accordingly, we used a collaborative co-constructive coding approach, reflecting on and discussing codes, categories, and themes among the co-investigators until consensus was reached.

As part of the semi-structured qualitative interviews, participants were also asked to rate their overall experience off the intervention on a scale from 0 to 10, where 0 indicated a completely negative experience and 10 a completely positive one. Furthermore, we also asked each participant to reflect upon their experience of the intervention/project and describe it using exactly three words. These words were coded openly and then merged into categories. The participants’ clinical data and SF-12 and rating scores were summarised using absolute and relative frequency. Descriptive statistics analyses were reported as absolute frequencies and percentages for categorical variables and as means and standard deviations (SDs) for continuous variables.

LE have been grouped in categories of system-based chronic and late medical health events (CLME), using the St Jude Lifetime Cohort Study (SJLIFE) modified version of the National Cancer Institute’s Common Terminology Criteria for Adverse Events (CTCAE) version 4.03. LE’ severity has been classified as mild (grade 1), moderate (grade 2), severe or disabling (grade 3), life-threatening (grade 4), or death (grade 5) [9].

## 3. Results

### 3.1. Quantitative Findings

Between January 2021 and January 2025, a total of 23 CAYAC survivors took part in the projects developed by UGI and Patchanka. Of these, 4 participants (17.4%) declined to participate in the follow-up study, 4 (17.4%) could not be contacted, 1 (7.7%) had not yet started the internship at the time of data collection, and 1 participant (7.7%) passed away due to complications related to late effects. The final sample therefore consisted of 13 participants.

The mean age-at-diagnosis was 12.94 years (SD 5,19; range 1–20) while the mean age-at-interview was 27.19 years (SD 5.29; range 21–40). Participants represented different diagnoses: Acute Leukaemia (Myeloid or lymphoblastic) (n = 5), Central Nervous System (CNS) Tumours (n = 4) (Medulloblastoma n = 2, Ependymoma, CNS Germ Cell Tumour), supplemented with singular cases of Ewing Sarcoma, Nasopharyngeal Rhabdomyosarcoma, Immature Teratoma, and Epstein–Barr Virus-Associated Hemophagocytic Lymphohistiocytosis.

A total of 40 late effects (LEs) were present, distributed, across all participants. Of these, 7 were grade 1 (17.5%), 25 were grade 2 (62.5%), and 8 were grade 3 (20%). Twelve out of thirteen patients (92.3%) experienced at least one LE, and the majority (9 out of 13) presented with multiple events, sometimes of different severity grades (Table 1).

The SF-12 results showed a mean PCS score of 45.2 (SD 9.1 range 33.7–64.3) and a mean MCS score of 43.5 (SD 11.2 range 26.3–63.1). Whereas, the average score on the 0–10 ranking reported by participants in their evaluation of the experience was 8.3 (SD 2.0 range 5.0–10.0) (Table 2).

### 3.2. Overall Experience and Three-Words Reflection Task

The participants rated their overall experience quite highly with a mean of 8.3 (SD: 2.0; min: 5, median: 9, max: 10) on a scale of 0 to 10, with only two participants rating it as 5 and the remaining rating it from 8 to 10. This rating is well-aligned with the analysis of results of the three-words reflection task.

We obtained a total of seven codes from all the words of the reflection task. These seven codes were then merged into three categories (rewarding, stimulating, challenging) as illustrated in Table 3.

**Figure 2:**
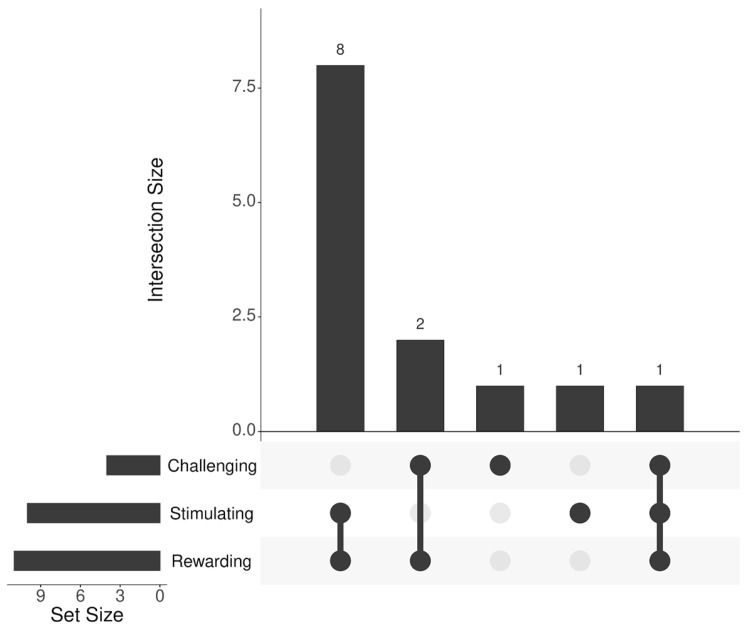
Distribution of categories to participants for the three-words reflection task.

The analysis of these three categories indicates that most of the participants experienced and perceived the intervention/project as rewarding (11 out of 13) and stimulating (10 out of 13). A minority (4 out of 13) described the intervention/project with words that indicate demanding (3 out 13) or limiting (1 out of 13) experiences. The distribution of the three categories from Table 3 regarding the number of participants that used included words, is visualised in Figure 2.

### 3.3. Results of the Thematic Analysis

The thematic analysis of our interview data gave rise to 20 categories, grouped into 8 themes, which we present in the following subsections. This presentation is accompanied by Table 4, which presents sample quotes for all the categories of each theme.

Theme 1: Motivations and initial expectations

Most participants reported having no specific expectations regarding the outcomes and trajectory of the project. Nonetheless, many were motivated to participate out of a desire for autonomy and financial independence (Category 1.1). Participants often approached the intervention with a strong desire to pursue employment and personal development (Category 1.2). However, many were facing their first work experience and expressed uncertainty about how to start or navigate the job market, especially given their physical impairments and medical history.

Theme 2: The impact of cancer on workability

Participants report that previous cancer—along with its related physical and psychological sequelae—has an impact on occupational choices (Category 2.1), influencing career goals and self-perceived workability. Physical impairments not only can decrease ability to work but, in some cases, can even lead to the loss of employment (Category 2.2). Moreover, previous cancer also modulates emotional well-being in professional settings (Category 2.3).

Theme 3: Accessibility and task adequacy

Some participants had previously undertaken job experiences independently, which were unfortunately not appropriate given their physical conditions and perceived as too challenging (Category 3.1). In contrast, the individualised approach of the intervention seems to facilitate the identification of workplaces that were both aligned with participants’ skills and compatible with their physical limitations (Category 3.2). This alignment was perceived to promote job stability. When asked whether the tasks assigned during the internship were appropriate for their physical conditions, the majority of participants responded with a strong affirmation: “Absolutely.”

Theme 4: The importance of building self-esteem and self-efficacy

Participants want to learn, build skills, be helpful, and feel integrated. The intervention experience appears to have influenced participants’ self-perception, fostering personal growth and enhanced self-awareness. This growth increases the participants’ self-esteem (Category 4.1), leaving them more confident. What is more, for most participants, the intervention also enhances self-efficacy (Category 4.2), allowing participants to gain the skills to operate more independently and self-reliantly. Several participants also experienced the intervention as helping them to move from pathology toward normality. As for many of the other participants, normality is associated with successful integration into a work environment, i.e., with occupational embeddedness (Category 4.3). Particularly the opportunity for some participants to have their internships extended or to be hired has helped enhance their self-perception.

Theme 5: The value of vocational support

Several participants highlighted the importance of individualised career assessment, particularly when aimed at enhancing general job skills (Category 5.1) such as the development of a curriculum vitae (CV). This process was often described as beneficial and, in many cases, as an experience never previously conducted with qualified professional coaching. The support through the intervention was perceived as impactful (Category 5.2) not only in identifying employment aligned with personal aspirations but also in discovering suitable yet engaging opportunities. The theatre and training phase was perceived by those who participated as particularly effective in enhancing professional and interpersonal skills, providing a focus on personal growth (Category 5.3).

Theme 6: The power of relationships: social support and workplace integration

Relational aspects of the work environment emerged as a key factor in participants’ positive experiences and successful integration, often through enhancing social interactions (Category 6.1). Support from tutors, colleagues, and customers not only enhanced participants’ self-efficacy and autonomy but also fostered a sense of belonging and motivation (Category 6.2). The intervention facilitated interaction with supervisors or managers at work and also with peers, which in some cases led to the formation of friendships, thereby promoting an expanded social network able to provide feedback and support (Category 6.3).

Theme 7: The double-edged impact of peer and family support

Participants frequently reported receiving consistent and encouraging support from family members, which positively influenced their engagement in project activities and daily routines (Category 7.1). However, this support is sometimes accompanied by a pattern of overprotection or persistent concern that CAYAC survivors may experience excessive fatigue, struggle to manage work responsibilities, or fear to fail coping independently (Category 7.2).

Theme 8: Critical aspects and the role of social support

While many participants reported positive outcomes, some expressed critical reflections on the professional impact of the intervention (Category 8.1). One key factor influencing the success of the intervention appeared to be the presence—or absence—of social support (Category 8.2). Participants who experienced stronger relational support reported greater clarity and confidence in their work integration process. In contrast, others struggled due to a lack of structured guidance. Notably, participant P1 had one of the least successful experiences in the programme, underscoring how insufficient support can hinder the development of self-efficacy and limit the collaborative, relational benefits that the intervention aims to foster.

## 4. Discussion

CAYAC survivors face significant challenges when entering the workforce, especially if they are affected by long-term physical LE or burdened by psychological issues. These factors affect not only their overall work ability, but also the capacity to perform specific tasks, whether physically demanding or cognitively complex. Also, participants’ health issues affect their approach to work, interactions with colleagues, and self-perception.

The job placement intervention described in this study has been developed in accordance with current survivorship care guidelines [7,21,24,26] and supported by recent evidence [3,11,15,18,20,34]. Participants were already enrolled in an integrated and multidisciplinary follow-up programme, where educational and occupational needs are routinely monitored alongside medical care [42,43]. The tailored approach where the intervention was implemented by a multidisciplinary team with vocational specialists playing a central role, effectively addressed a key challenge identified in the semi-structured interviews: the difficulty not only in finding work but in identifying suitable and accessible job opportunities. Many participants were at the beginning of their professional journey or facing their first real work experience, a stage that can be particularly complex for survivors due to health-related barriers and limited prior exposure to the labour market [3,34]. By providing personalised counselling and practical experience, the intervention helped survivors overcome employment barriers and access meaningful work. This approach is consistent with the findings of Pole et al. [34], who demonstrated that individualised goal-setting within vocational counselling enhances motivation, tracks progress, and facilitates successful workforce integration.

Clinical complexity was high across participants. Twelve out of 13 (92.3%) survivors reported at least one LE, most of moderate severity. Moreover, 9 out of 13 participants were officially recognised as having a physical, cognitive, or psychological disability according to the criteria defined by the Italian legal framework [44,45] (Law 104/1992; Law 68/1999). In Italy, disability certification is issued by public medical committees and based on documented impairments and functional limitations, and serves to guarantee access to social, educational, and occupational support [46]. Although country-specific, the classification broadly reflects the biopsychosocial model outlined by the World Health Organization’s International Classification of Functioning, Disability and Health [47].

Despite the complex medical history, HRQoL scores remained generally within normative ranges, indicating a certain level of functional adjustment. However, in the SF-12 questionnaire the lower average Mental Component Score (MCS = 43.5) compared to the Physical Component Score (PCS = 45.2) may indicate that psychological and emotional issues are more impactful than physical limitations. Many participants expressed anxiety and concerns in interviews about how their health limitations might interfere with their ability to work. This pattern is consistent with prior studies documenting the long-term psychosocial impact in adolescent and young adult cancer survivors [48].

Notably, several participants emphasised that their current difficulties were not primarily linked to their oncological history but to ongoing physical constraints that hinder access to employment opportunities matching their true capabilities. Our findings are consistent with the evidence that the cumulative burden of chronic health conditions—particularly neurologic and sensory impairments—can be linked to unemployment and can further compromise employment stability. Given the early onset of multiple morbidities, targeted clinical and vocational support is essential to reduce long-term workforce exclusion [25]. Other studies [3,25,49,50] similarly observed that physical and mental impairments related to cancer and its treatment affected cancer survivors’ work ability, the capacity to keep up with work demands, and employment status. To address these challenges and support long-term functioning, adopting more flexible working conditions (e.g., adjusted schedules, opportunities for remote engagement, and guidance from occupational health specialists) may play a key role in improving quality of life among survivors [3,15,51]. In our sample, several participants reported benefiting from tasks that were well aligned with their functional needs, and the flexibility offered by internship programmes facilitated engagement and reduced strain.

The primary motivation for joining the programme was the desire for greater independence, particularly financial autonomy, a universal goal at this life stage, that can be particularly challenging. This finding is consistent with existing literature [17,19,25,33], which highlights financial independence as a central concern for young adult cancer survivors navigating the transition to adulthood.

The intervention was generally well appreciated, with participants reporting high overall satisfaction (mean score 8.3/10). Most of them described the experience as rewarding and stimulating, indicating that it was both meaningful and empowering. However, a few participants also found it demanding or limiting, suggesting the need for flexibility and individualised pacing to accommodate different capacities and expectations. Moreover, the time restricted duration of internships may represent a significant limiting factor, especially for individuals whose contracts are not renewed. Such employment discontinuity contributes to long-term economic instability [25].

One of the most important resources reported was social support, provided by family, tutors, and colleagues. Social support may reduce the risk of work withdrawal or early retirement by enhancing self-efficacy, which has been shown to mediate this relationship [52]. While social support can be a key protective factor [14,27,33], it may also present challenges. Overprotection, particularly from parents, can hinder the development of autonomy and self-efficacy [4] unless balanced with appropriate encouragement and expectations. Many participants noted this ambivalence, highlighting the need to educate families about promoting independence in survivors, even when health concerns persist. This balance between emotional support and autonomy becomes even more delicate in the presence of complexities or disabilities, as observed in the analysed sample, where survivors may require additional assistance but still need opportunities to foster self-reliance and personal growth.

Finally, the intervention had a positive impact on participants’ self-perception, enhancing their sense of competence, responsibility, and confidence in managing tasks such as internships and contract renewals, all of which represent core elements of adult identity and workforce integration. These findings are consistent with those reported by Maas et al. [13], who demonstrated that individual perceptions, illness-related beliefs, self-esteem, and social support significantly influence long-term emotional, social, cognitive, and physical well-being in survivors. Their study showed that these psychosocial factors have a greater impact on psychosocial functioning than socio-demographic or medical characteristics alone. Together, these results highlight the value of targeted interventions aimed at fostering self-acceptance, strengthening self-esteem and social support. They also address feelings of helplessness and negative self-perception, with the ultimate goal of improving survivors’ overall quality of life.

This study has several limitations that should be acknowledged. The limited sample size, clinical heterogeneity, and single-centre design restrict the generalisability of the findings and limit the possibility of conducting robust statistical analyses. Additionally, the variability in internship experiences among participants complicates the identification of specific factors driving the observed outcomes. When evaluating social outcomes such as unemployment, it is crucial to consider the Italian cultural and socio-economic context (REF), where youth unemployment rates and the structure of labour and welfare systems may have influenced both participation and perceived benefits. Furthermore the sustainability and potential cost-effectiveness of this type of structured, multidisciplinary intervention were not assessed and should be addressed in future studies, as resource requirements may affect feasibility in routine survivorship care.

Nonetheless, there is a paucity of research investigating structured vocational interventions within this population. Most existing studies focus primarily on occupational challenges and associated risk or protective factors without evaluating the effectiveness of targeted support programmes. Despite its limitations, this study contributes preliminary evidence supporting the feasibility and potential benefits of individualised vocational interventions that can be closely integrated into cancer survivorship programmes. The transferal of our experience out of the Italian context, based on a tailored and specialist-guided approach, may require a deep effort in adapting interventions to different socio-cultural and economic contexts. Designing and conducting studies exploring the feasibility of this approach across other countries might generally represent a challenge for health care providers and stakeholders involved in survivorship care programmes. The subsequent dissemination and critical revision of these practices and experiences may further contribute to optimise their applicability in different health care systems.

## 5. Conclusions

This pilot study provides preliminary insights into the feasibility and perceived value of a tailored vocational intervention for CAYAC survivors. The findings suggest that structured, multidisciplinary approaches may support the transition to employment and merit further exploration. Larger and more diverse samples, together with longitudinal designs, are needed to evaluate the efficacy of vocation programmes in addressing the employment needs of young cancer survivors.

## Figures and Tables

**Table 1:** Socio-demographic and medical characteristics of participants.

	No.	%
**Sex**		
Female	6	46.2
Male	7	53.8
**Age at diagnosis**		
0–4	2	15.4
5–9	0	0.0
10–14	6	46.2
≥15	5	38.4
**Age at the interview**		
20–24	5	38.4
25–29	6	46.2
30–34	1	7.7
≥35	1	7.7
**Off-therapy**		
<2000	1	7.7
2000–2009	2	15.4
2010–2014	4	30.8
≥2015	6	46.2
**Education**		
Middle school	4	30.8
High school	8	61.5
University	1	7.7
**Diagnosis**		
Acute Leukaemia (Myeloid or lymphoblastic)	5	38.4
CNS Tumours	4	30.8
Others *	4	30.8
Chemotherapy	12	92.3
Radiotherapy	6	46.2
Neurosurgery	4	30.8
Hematopoietic stem cell transplantation	2	15.4
Disease Relapse	1	7.7
Late effects **		
Patients with ≥ 1 LE	12	92.3
Grade 1	7	17.5
Grade 2	25	62.5
Grade 3	8	20.0
Second Neoplasm	2	15.4

* Ewing Sarcoma, Immature Teratoma, Nasopharyngeal Rhabdomyosarcoma, and Epstein–Barr Virus-Associated Hemophagocytic Lymphohistiocytosis. ** According to the National Cancer Institute’s Common Terminology Criteria for Adverse Events (CTCAE) version 4.03.

**Table 2:** SF-12 physical and mental health scores and 0–10 self-reported ratings of perceived satisfaction with the intervention.

	M	SD	Range
SF-12			
PCS	45.2	9.1	33.7–64.3
MCS	43.5	11.2	26.3–63.1
0–10 rating	8.3	2.0	5.0–10.0

**Table 3:** Codebook of the three-words reflection task.

Codes	Beneficial	Satisfying	Relational	Educational	Stimulating	Limiting	Demanding
Categories	rewarding			stimulating		challenging	

**Table 4:** Sample quotes for all 20 categories grouped by the 8 themes.

Theme 1: Motivations and Expectations	
Category 1.1: Desire for autonomy and financial independence	Hoping to get hired and have a salary. (P1) The desire to leave my home and feel more independent (P2; P9)—to be useful to others. (P2) I wanted to be independent, to stop asking my parents for money. I wanted to work! (P3)
Category 1.2: Desire for employment and personal development	I didn’t have expectations about what I’d like to do, but they formed over time through these experiences. (P4) I made mistakes. Since it was my first real job, I didn’t behave at my best. (P6) I didn’t even know where to start looking for a job. (P6; P8) I was willing to do anything, within my limits. (P8) The desire to work, grow, learn new things, and to feel normal in a way. After being in the hospital context, doing heavy jobs isn’t ideal. (P12)
Theme 2: The impact of cancer on workability	
Category 2.1: Physical constraints on occupational choice	My career goals have changed a little bit. The doctor told me that certain jobs would be too demanding for me and my health, and suggested that office work would be better for me. (P2) I realized that the problems I had in the past are still affecting me: fatigue and physical health issues. I’ve always avoided and denied being limited by my illness, but then I realized I do have limitations. I’m cancer free, but I get tired very easily. (P3) I would have loved to attend a sports-focused high school, since I was really passionate about sports. Unfortunately, due to my physical conditions, I was told it wasn’t possible. (P5) I do have eye-related issues, and I think that it would affect any job. (P13)
Category 2.2: Employment loss due to physical impairments	Some jobs are hard if you are not healthy. I had to stop [a previous job] because of severe shoulder pain. They needed someone who could function every day. (P3) I had to stop [my internship] due to a surgery that kept me in the hospital for a month. After that they weren’t able to keep me on. (P5)
Category 2.3: Impact on emotional well-being	I always have anxiety about feeling unwell at work. (P3) A lot of us have our own struggles. (P7) Most things I’ve faced aren’t comparable to what I went through in the hospital—nothing was harder than that! (P12)
Theme 3: Accessibility and task adequacy	
Category 3.1: Previous work-related challenges	I found a job on my own as a construction worker for about a year, but it was very hard. Even though I earned money, I chose to quit because I wasn’t well. (P10) The shift working hours can be tough for someone with a medical past, even if it’s not physically demanding. I’d already worked in physically demanding jobs before, so I wanted to change the environment a bit. (P12)
Category 3.2: Successful alignment with workability	They did their best to arrange the internship. (P3) They had informed me that I’d be working at the register due to my physical limitations. Occasionally, I took on other tasks, but nothing too physically demanding. (P5) [The intervention] helped me find something more stable [work]. (P7). Now I sit at a desk and don’t have to exert myself physically. (P13)
Theme 4: The importance of building self-esteem and self-efficacy	
Category 4.1: Increasing self-esteem	I suffer a lot from anxiety—especially when I’m around people, or when I have to talk to many people at once, or juggle a lot of tasks. This job helped me manage that. (P5) Before, I saw everything negatively—like I wasn’t good at anything. Now, even if a [job] interview doesn’t go well, I treat it as experience and not as failure. (P8) [The intervention] was short, so I’m not sure about a full self-esteem shift, but it gave me more confidence to try things on my own. (P9) I didn’t imagine myself wearing a shirt every day. It’s a formal environment. My mindset has really changed. (P12)
Category 4.2: Enhancing self-efficacy	Now, that I’ve had a serious experience, I understand how the work world operates—how to behave with colleagues, how to collaborate with them and with managers. (P2) Now, I see my future in a more positive and autonomous way. I am definitely more autonomous in terms of work tasks. (P4) I’ve built up my skills, I feel confident and independent. I no longer need assistance. I developed skills, learning how to adapt different situations. (P5) I’ve changed for the better. Now I enjoy working, I’m active, my days are fuller, and I have a daily routine. (P10)
Category 4.3: Occupational embeddedness	I am very happy because they asked me to stay. Now I also work night shifts. It’s more demanding and I have more responsibilities. (P5) My supervisor wants to offer me a one-year contract. (P6) Especially now that they’ve offered me a contract, I know that a company might actually want me. (P13)
Theme 5: The value of vocational support	
Category 5.1: Enhancing general job skills	The CV writing session was really helpful—mine needed a lot of work—and [the career coaches] helped me in improving it. (P7) It was my first time. They help you to understand and guide you in what you might want to do, your options, the pros and cons. They helped me think about other paths, other possibilities through my CV. (P12) I’d written a CV before, but something was missing, so they helped me to structure it better. (P13)
Category 5.2: Perceived impact of the intervention	The project helped by connecting me with the right people and finding options that suited me. (P8) The tasks of the internship were aligned with what we had discussed during the individual interview. During the second interview, I got the chance to do an internship—that made it feel more concrete. The project helped me narrow things down and find what to specialize in. (P9)
Category 5.3: Focus on personal growth	It was definitely a stimulating experience. It helped me work through the anxiety I feel when speaking in front of others, facing large audiences, and juggling several tasks simultaneously. (P5)
Theme 6: The power of relationships: social support and workplace integration	
Category 6.1: Growth through social interactions	Interacting with customers helped me break out of my shell. I used to be shy and awkward, but I really came to enjoy those interactions. Now, I’m much more confident in conversations with people. (P8) The most pleasant moment was when they trusted me enough to leave me in charge of the structure. Working with people and being in contact with them, it’s fulfilling. (P12)
Category 6.2: Sense of belonging and motivation	My tutor used to stop by the office every morning and say that even if there was nothing to sign, she wanted to see my smile—it made her day better. (P2) I got along even better with my last manager than the previous one. She really taught me a lot, and I became very patient. (P3) One colleague in particular still offers help—even now that I’m working night shifts, I know I can rely on her if anything comes up. (P5) I felt supported and comfortable whenever I needed help. I think that finding good people at work makes a huge difference. One of the girls from Patchanka advocated for me, and that helped get my contract extended. (P7) My tutor is very kind and helpful, but she lets me handle things independently, too. She gives me general guidance and says ‘Go ahead, and I’ll review it.’ (P13)
Category 6.3: Peer feedback and support	I also made friends during the project, and I still keep in touch with them. I met a girl who became my best friend. (P5) Also, group activities taught me a lot about myself. I picked up on things I hadn’t noticed before, especially through feedback from others and moments shared during group work. I became especially close with my tutor. I also learned tailoring from a colleague, and we’re still friends. (P8)
Theme 7: The double-edged impact of family support	
Category 7.1: Family support as a facilitator	[Family support] helped very much. They were happy because they saw I was happy, too. (P2) I used to complain a lot, and my family encouraged me to join. They were very supportive. (P8) My family supported me, both emotionally and practically, like giving me rides when they can. They supported and motivated me throughout. (P9) Yes, my family has always supported me in general and also encouraged me to continue with these projects. Even during the tough times, my mother gave me strength. From when I got sick until now, she’s always stayed close to me. (P11)
Category 7.2: Family support as a barrier	They weren’t against it, but when I wasn’t feeling well, they advised me to stop if I wasn’t okay. (P3) My mom always worries I might be too tired, so I try to reassure her. Previous jobs were short and poorly paid. My brother wasn’t thrilled, but for me it was nevertheless a way to get out of my home. (P7)
Theme 8: Critical aspects and the role of social support	
Category 8.1: Critical reflections	I thought it would be more helpful in finding a job. It was useful as a personal experience—but not professionally. (P11)
Category 8.2: Importance of social support at the workplace	There wasn’t a specific person explaining things, so I asked different people in the stores. (P1) What truly made a difference was having someone help me at the beginning to understand what I needed to do. (P2)

## Data Availability

The data presented in this study are available on request from the corresponding author.

## Abbreviations

The following abbreviations are used in this manuscript:

CAYAC	Childhood, adolescent, and young adult cancers
CNS	Central nervous system
RTW	Return to work
SWTP	School and Work Transitions Program
HRQoL	Health related quality of life
SF-12	12-Item Short Form Health Survey
PCS	Physical Component Summary
MCS	Mental Component Summary
LE	Late effect
CLME	Chronic and late medical health events
CTCAE	National Cancer Institute’s Common Terminology Criteria for Adverse Events
CV	Curriculum Vitae
WHO	World Health Organization
ICF	International Classification of Functioning, Disability and Health
